# MGL/CLEC10A is an important C-type lectin receptor activated in the innate immune response to *Mycobacterium tuberculosis* and is suppressed in people with HIV

**DOI:** 10.3389/fimmu.2025.1597281

**Published:** 2025-10-01

**Authors:** Sarah E. Browning, Preeti Bharaj, Xiuzhen Fan, Harrison Nguyen, Alex J. Holloway, Kubra F. Naqvi, Joshua G. Lisinicchia, Reina N. Paez, Sadhana Chauhan, Matthew B. Huante, Yazmin B. Martinez-Martinez, Mark A. Endsley, Benjamin B. Gelman, Janice J. Endsley

**Affiliations:** ^1^ Department of Microbiology and Immunology, University of Texas Medical Branch (UTMB), Galveston, TX, United States; ^2^ Department of Pathology, UTMB, Galveston, TX, United States

**Keywords:** tuberculosis, human immunodeficiency virus, C-type lectin receptor, macrophage galactose-type lectin (MGL), CLEC10A, innate immunity

## Abstract

*Mycobacterium tuberculosis* (Mtb) and HIV are leading infectious causes of death worldwide and act synergistically to worsen disease during co-infection. C-type lectin receptors (CLR) respond to pathogen-associated carbohydrates to activate downstream innate immunity and can be exploited for entry of intracellular pathogens. The macrophage (MΦ) galactose-type lectin (MGL, CLEC10A) is an immunomodulatory CLR associated with M2 MΦ. We previously described an immune role for a murine MGL homologue in an experimental model of tuberculosis (TB). Herein we extend these findings by identifying human MGL as an important member of the Mtb-responsive pathogen recognition receptor (PRR) repertoire that is activated in both M1 and M2 polarizing conditions. MΦ exposure to Mtb activates MGL expression and abundant MGL+ cells are present in TB granulomas of human lung and lymph node. Silencing of MGL permits greater Mtb replication in MΦ derived from human peripheral blood monocytes. Compared to healthy controls, MΦ and neutrophils of people with HIV (PWH) have reduced MGL and tissue MGL levels negatively correlate with viral load. Binding assays with recombinant MGL demonstrates direct interaction with Mtb, but not HIV. *In vitro* Mtb exposure of PBMC from PWH revealed potential recovery of the MGL defect as well as a differential activation of MGL compared to the DC-SIGN and MR CLRs. MGL is thus an important mechanism of innate immunity and potential target for host directed therapy in those with TB or TB-HIV.

## Introduction

TB has re-emerged as the leading cause of infectious disease death following the decline of COVID-19 mortality (1). Efforts to identify immune mechanisms that can be targeted through host directed therapies or vaccine strategies are key to reducing the 10.8 million infections and 1.25 million deaths per year due to TB (2). Co-infection with Mtb and HIV is a related public health crisis that is often described as a “syndemic” because of the reciprocal relationship between these pathogens (3, 4). An HIV positive status is the largest risk factor for developing TB, and PWH have a higher prevalence of latent TB reactivation, drug treatment failure, or development of drug-resistant TB (3, 5, 6). TB mortality disproportionately affects PWH and the leading cause of death among this population, constituting almost one-third of HIV/AIDS deaths (5, 7). In PWH, a superimposed Mtb infection is also associated with poorer clinical outcomes of HIV. Activation of the immune system by Mtb promotes replication of HIV, accelerating lymphocyte depletion (3, 4). The introduction of antiretroviral therapy (ART) for treatment of HIV was a major milestone that significantly reduced TB-related deaths in PWH. Despite these advances, PWH who are taking ART remain at an increased risk of developing TB due to both drug and infection associated immune defects (3). Efforts to augment immunity to Mtb must therefore account for HIV-mediated impairment of host defenses including in those taking ART.

MΦ are a secondary host and reservoir for HIV infection, as well as the primary cellular niche for Mtb (8, 9). HIV infection is known to compromise MΦ anti-mycobacterial immunity through incompletely understood mechanisms that include reduced antigen presentation and defective intracellular killing (10–12). MΦ and other myeloid cells identify and respond to pathogens through germline encoded pattern recognition receptors (PRR) that recognize conserved molecular patterns and structures (13). The PRRs families include toll-like receptors (TLR), NOD-like receptors (NLR), and C-type lectin receptors (CLR). CLRs constitute a large family of PRRs that bind carbohydrate structures in a calcium-dependent manner (14). Most CLRs are membrane-bound and their activation triggers endocytosis. The subsequent antigen processing and presentation make these receptors critical for communication between the extracellular environment and the immune system (15). HIV and Mtb are both pathogens that can exploit CLRs and PRR systems to gain entry or alter MΦ function. Mtb infects and replicates within MΦ, and different CLRs have been implicated at various steps during the pathogenesis of TB (16). CLRs, such as dendritic cell-specific ICAM-grabbing non-integrin (DC-SIGN) and Mannose Receptor (MR), are known to play a role in HIV pathogenesis as well. These receptors recognize HIV-1 and allow attachment of the virus to myeloid cells which promotes trans-infection of T lymphocytes (17–19). A greater understanding of the CLR repertoire that determines innate immune responses to Mtb and HIV is thus critical to understanding both mono- and co-infections with these pathogens.

In a model of experimental tuberculosis, our laboratory previously identified a host immune role for a murine homologue of the MΦ galactose-type lectin (MGL) receptor (also known as CLEC10A or CD301) (20). MGL is a C-type lectin receptor expressed mainly on MΦ and dendritic cells that recognizes terminal galactose and N-acetylgalactosamine (GalNAc) residues (21). MGL is the only receptor on antigen presenting cells (APC) known to bind GalNAc residues and interacts with both self and non-self-antigens such as O-glycans to modulate immune responses (22–24). Whereas humans express a single MGL receptor, mice express two non-redundant receptors, MGL1 (CD301a) and MGL2 (CD301b). It is currently unknown which characteristics are shared among these three homologues, though evidence has demonstrated that the function of human MGL may be differentially executed by mouse MGL1 and MGL2 (25). This receptor also contributes to antigen processing and presentation by MHC-1 and MHC-II to CD8+ and CD4+ T cells, making it an important bridge between innate and adaptive immunity (26, 27).

Limited studies to date indicate an important contribution of MGL in myeloid cell host defense against human pathogens. In *Mgl1^-/-^* mice infected with Mtb, bacterial burden and pulmonary inflammation was significantly increased compared to wild-type littermates, suggesting an active role in bacterial clearance (20). MGL1 is also needed for resolution of pulmonary inflammation in a mouse model of gram-negative pneumonia due to infection with *Klebsiella pneumoniae* (28). Loss of MGL1 in this model led to greater pulmonary neutrophilia and tissue destruction in the absence of changes in bacterial burden. *Mgl1^-/-^* MΦ infected with *Trypanosoma cruzi* had impaired production of TNF and IL-12 and failed to activate NF-κB (29). Taken together, these studies demonstrate that MGL mediates important antimicrobial and immunomodulatory effects and highlight its importance in innate immunity. The emerging role of MGL in host immunity to cancers and infectious disease as well as immune homeostasis is the basis for several on-going efforts to understand ligand recognition and exploit for host directed therapies (30–34).

In the present study, we identify a direct pathogen receptor recognition for the CLEC10A/MGL CLR in response to Mtb infection. Relevant to TB and HIV co-infection outcomes, assessment of cells and tissues from PWH further demonstrates HIV-related defects in MGL pathways including in macrophages, neutrophils, and lung tissue. MGL displays a differential response to Mtb compared to DC-SIGN and MR and can be activated by Mtb antigen in PBMC of PWH. Overall, these results suggest MGL is an important mechanism of innate immunity against Mtb that is susceptible to disruption by HIV and may offer opportunities for therapeutic intervention.

## Methods

### Sex as a biological variable

Analysis included use of de-identified samples from a regional blood bank and information on sex of different source donors was not available. Some experiments were performed using samples from male donors only due to lack of sufficient female donors in the biorepository. The samples were provided by the National NeuroAIDS Tissue Consortium (NNTC), whose cohort is approximately 81% male. These results may thus have limitations in their applicability to female cohorts.

### Cell culture and MΦ differentiation

Human PBMC isolated from the buffy coats of de-identified donor blood samples were obtained from the Gulf Coast Regional Blood Center (Houston, Texas). PBMC were isolated via gradient centrifugation on Accuprep, washed, and cultured in RPMI media supplemented with 10% FBS, 1% pen-strep, and 1% L-glutamine (cRPMI). Cryopreserved human PBMC from HIV+ and age matched HIV- donors were obtained from the NNTC at UTMB in Galveston, TX under Institutional Review Board (UTMB IRB) number 98-402. Samples were thawed in a 37°C water bath and immediately transferred to pre-warmed RPMI supplemented with 20% FBS. Cells were isolated by centrifugation, resuspended in cRPMI supplemented with 20% FBS, and placed in a 5% C02 incubator for a 2 h recovery period prior to use. Monocyte-derived MΦ (MDM) were generated by culturing PBMC in tissue culture treated flasks and allowing monocytes to adhere overnight. Non-adherent cells were subsequently discarded, adherent cells were removed using cell disassociation buffer (Sigma), counted, resuspended at 5x10^5^ cells per well in 12 well plates, and MΦ were derived by 5–7 days of incubation with 25 ng/mL recombinant human M-CSF (Peprotech) in cRPMI. In other experiments, whole blood from HIV+ or HIV- donors was used for analysis of MGL expression by neutrophils.

### Use of human tissue

Human autopsy tissue from decedents with HIV were obtained from the NNTC for use in this study. Autopsy and biopsy tissue were also obtained from UTMB Autopsy service archives of formalin-fixed paraffin embedded tissues.

### Bacterial culture

*M. bovis* BCG and *M. tuberculosis* H37Rv were obtained from the ATCC and maintained in liquid culture in Middlebrook 7H9 media (Difco) supplemented with 10% OADC (BBL), 0.5% glycerol (Fisher Scientific), and 0.05% Tween 80 (Fisher Scientific). Cultures were handled in accordance with institutional biosafety requirements. Concentration of bacteria was calculated by measuring the optical density of cultures at 600 nm (OD_600_) and using a conversion rate of 3x10^8^ CFU/mL at an OD_600_ of 1.0. Cultures were maintained and used for infections at mid-log phase (OD_600_ = 0.6).

*E. coli* F470 and *E. coli* F653 were generously supplied by Professor Gerald Koudelka from the University of Buffalo with the permission of Dr. Chris Whitfield at the University of Guelph who was the original source of the strains. Glycerol stocks of each bacterial strain were streaked onto LB agar plates and incubated overnight to isolate single colonies which were then inoculated into LB broth and incubated overnight in an orbital shaker at 200 rpm. Concentration of bacteria was calculated by CFU enumeration of 10-fold serial dilutions of liquid culture.

### MΦ stimulation and infection

Cytokine stimulation of cells was conducted in cRPMI conditioned with 20 ng/mL IL-4 or IL-10 for 24 h before harvesting for flow cytometry. Infections with BCG or Mtb were performed by adding the appropriate number of mycobacteria to achieve 5 or 10 MOI, resuspended in PBS, to cell cultures for 24 h prior to downstream applications.

### Flow cytometry

Flow cytometry was used to detect expression of MGL and other CLRs in PBMC and MDM following experimental treatment or infections. Whole blood staining for neutrophils was performed on samples collected and stored at room temperature. Cells were washed with PBS and incubated with a viability dye (Fixable Near-IR, Invitrogen) in PBS for 10 min at 4 °C and washed with FACS buffer composed of PBS, 1% (w/v) bovine serum albumin (Fisher), and 0.1% sodium azide. Before extracellular staining, cells were incubated with anti-CD16/CD32 Fc block reagent (BD Biosciences) for 5 min at room temperature to reduce non-specific binding. Surface expression of various cell markers was detected by incubating cells with the appropriate antibodies for 1 h at 4 °C. Unbound antibody was removed by wash with FACS buffer and cells were fixed by resuspending in 4% ultra-pure formaldehyde for 48 h to inactivate pathogens. Samples were acquired by using a BD LSRFortessa in the UTMB Flow Cytometry and Cell Sorting Core Facility followed by downstream analysis using FCS Express version 6 (*de novo* software) or FlowJo v10. Side scatter and forward scatter characteristics were used to select leukocytes, and viable, single cell events were further isolated by selective gating to exclude doublets and cells permeated with live/dead NIR reagent. Cell populations were identified by surface phenotype consistent with monocyte, macrophage and neutrophil cells including CD14 (PE Vio770, Miltenyi Biotec, clone: TUK4), CD16 (V500, BD Biosciences, clone: 3G8), mature macrophage marker (eFluor 660, eBioscience, clone: 25F9), CD33 (BUV 496, BD Biosciences, clone: P67.6) and CD15 (BUV 395, BD Biosciences, clone: HI98). C type lectins were detected with antibody to MGL (Alexa Fluor 405, R&D Systems, clone: 744812), DC-SIGN (Alexa Fluor 594, R&D Systems, clone: 120507), and MR (PerCP/Cy5.5, BioLegend, clone: 15-2).

### MGL silencing

To silence MGL expression in primary MDM, siRNA complementary to *Clec10A* mRNA (Dharmacon) or a non-specific siRNA control, was transfected into the cells using Lipofectamine 2000 (Invitrogen) for 4–6 h according to manufacturer instructions. To demonstrate silencing, cells were harvested 24–96 h post-transfection to assess gene and protein knock down. For bacterial killing assays, the cells were infected with 10 MOI Mtb (H37Rv) 48 h after transfection. Cells were lysed 24 h post infection and serial dilutions of lysates were grown on 7H11 media for 3 weeks to quantify CFU.

### Immunohistochemistry and immune fluorescence

Human tissue sections from lung or lymph nodes collected during autopsy or for surgical pathology assessment were formalin-fixed and embedded in paraffin prior to staining. Tissue sections were deparaffinized and rehydrated by submerging in xylene followed by decreasing concentrations of ethanol. Samples were then submerged in PBS to fully rehydrate tissue.

For IHC, antigen retrieval was performed by submerging slides in citrate buffer pH 6 and heating to 95-100°C for 20 min in a vegetable steamer (Oster). After cooling, slides were washed in PBS with 0.05% Tween 20 (PBST). Samples were blocked to decrease non-specific staining by incubating tissue with goat serum for 1 h followed by Bloxall (Vector Laboratories) for 10 min. Tissue were then incubated in a humidity chamber with monoclonal antibodies targeting MGL (Abcam, 1:500) overnight at 4°C. After incubation, sections were washed with PBST followed by incubation with secondary antibody included in the Vectastain ABC-AP kit (Vector Laboratories) for 1 h at room temperature. Positive binding was visualized with ImmPACT Vector Red AP substrate kit (Vector Laboratories) followed by counterstaining with Mayer’s Hematoxylin.

For IF, antigen retrieval was conducted by submerging slides in antigen retrieval buffer (Perkin Elmer) for three 5 min intervals in a microwave specialized for tissue processing (BioGenex EZ-Retriever) to maintain temperature at 95°C for a total 15 min. After cooling, slides were washed with TBS with 0.05% Tween 20 (TBST). Samples were blocked to decrease non-specific staining by incubating tissue with antibody diluent/block (Perkin Elmer) for 10 min. Tissue were then incubated in a humidity chamber with monoclonal antibodies targeting MGL (Abcam, 1:10) overnight at 4°C. After incubation, sections were washed with TBST and once again blocked with antibody diluent/block for 10 min followed by incubation in a humidity chamber with goat anti-rabbit secondary antibody (Alexa Fluor 555, Invitrogen) for 2 h at room temperature. Excess antibody was washed with TBST followed by staining with DAPI (Invitrogen) and positive markings visualized by epifluorescence microscopy.

### Western blot

Frozen tissue samples were homogenized in 7x volume of buffer (10 mM Tris–HCl, 5 mM MgCl_2_, 0.5 mM dithiothreitol (DTT), 0.1% Triton X-100, pH 7.8) in the presence of protease inhibitors (Sigma) by silica bead beating and sonication. Samples were then spun in a centrifuge at 15K RPM for 10 min. Protein concentration was determined using Bio-Rad Protein Assay (Bio-Rad Laboratories) and bovine serum albumin (BSA) standards. 30 ug of total protein were loaded into Criterion Precast 18-well 4–20% gradient Tris–HCl gel (Bio-Rad Laboratories) for SDS-PAGE electrophoresis and run at 180 volts for 45 min. Separated proteins were transferred to Immobilon FL PVDF membrane (Millipore Sigma) in 10 mM Tris-glycine buffer containing 10% methanol at 100 volts for 1 h at 4 °C. The membrane was blocked in Intercept Blocking Buffer (LI-COR Technology) for 1 h and incubated with rabbit anti-CLEC10A (Abcam) or mouse anti-GAPDH (Santa Cruz Biotechnologies) primary antibodies diluted in blocking solution overnight at 4 °C. The membrane was washed three times in TBST and incubated with IRDye 800CW Goat anti-Rabbit (LI-COR) diluted in blocking solution for 1 h. The membrane was scanned with Odyssey Fc Imager (LI-COR) for 2 min. Band densities were measured using Image Studio Ver 4.0 (LI-COR).

### Multiplex ELISA

Supernatants from human PBMC that were cultured *in vitro* with Mtb or mock (PBS) were removed after 24 h of stimulation and frozen at -80°C. Subsequently, frozen samples were irradiated on dry ice for a total of 5 MRAD to inactivate pathogens using a cesium irradiator. Activation of cytokines and chemokines by Mtb in HIV+ and HIV- PBMC were determined by using a human Bioplex ELISA (BioRad) per manufacturer instructions. Quantity of cytokines and chemokines was estimated relative to a standard curve generated with included standard controls. Values determined to exceed the range (out of range high) were set to the value of the highest standard.

### MGL binding assays

Binding assays were performed using a recombinant antibody with Fab regions constructed to express human MGL amino acids 61 through 316 (Abcam). Mtb (H37Rv) was grown to log phase (OD_600_ = 0.6) and *E. coli* (F470 and F653) were grown overnight. For bacterial binding assays, 2-4x10^8^ CFU were labeled with CFSE by 45 min incubation with 10 uM CFSE and washed with PBS. Labeled bacilli were then incubated at 37°C with 2 μg of the rMGL-Fc for 45 min and washed with PBS. Positive binding was determined with a PE-conjugated anti-Fc antibody (Invitrogen) for detection via flow cytometry. For the HIV binding assay, protein G Dynabeads (Invitrogen) were incubated with 4 ug of the rMGL-Fc for 15 min to coat the beads, followed by incubation with 2-5x10^4^ infectious units of HIV_ADA_ for 45 min at 37°C. This assay exploits the strong binding between protein G and the Fc of the recombinant molecule to determine MGL capture in the absence of a candidate viral moiety. Binding was detected by immunoprecipitation followed by qPCR for HIV *gag*. RNA was extracted from virus using Quick-RNA Microprep Kit (Zymo) and cDNA was synthesized using the BioRad iScript cDNA Synthesis Kit. Samples were prepared for qPCR using the BioRad iTaq Universal SYBR Green Supermix and gene expression fold changes determined in reference to virus stock. *gag* forward: GGAAGCTGCAGAATGGGATA, *gag* reverse: GCTATGTCACTTCCCCTTGG. rMGL-Fc pre-incubated with 10 mM EGTA for at least 2 min served to establish background signal when calcium-dependent MGL binding is inhibited.

### Statistical analyses

Data analyses and graphical presentations were performed by use of GraphPad Prism 10 software (La Jolla, CA). Results are shown as mean ± SEM, and statistical analysis of data with multiple groups was performed by using one-way ANOVA followed by the two-stage Benjamini, Krieger, and Yekutieli procedure to control false discovery rate (FDR) for multiple comparisons. Significance was considered with any value with an FDR < 0.05, and a statistical trend was considered for any value with an FDR < 0.1. A two-tailed Students T-test was used to determine significant differences in experiments comprised of only two treatment groups. A Pearson’s Correlation Coefficient was utilized to determine statistically significant relationships between continuous variables. Significance was considered with any p value <0.05, and p values of 0.1 were considered a statistical trend.

## Results

### MGL expression by human MΦ is activated by mycobacteria and contributes to antibacterial function

**Figure 1:**
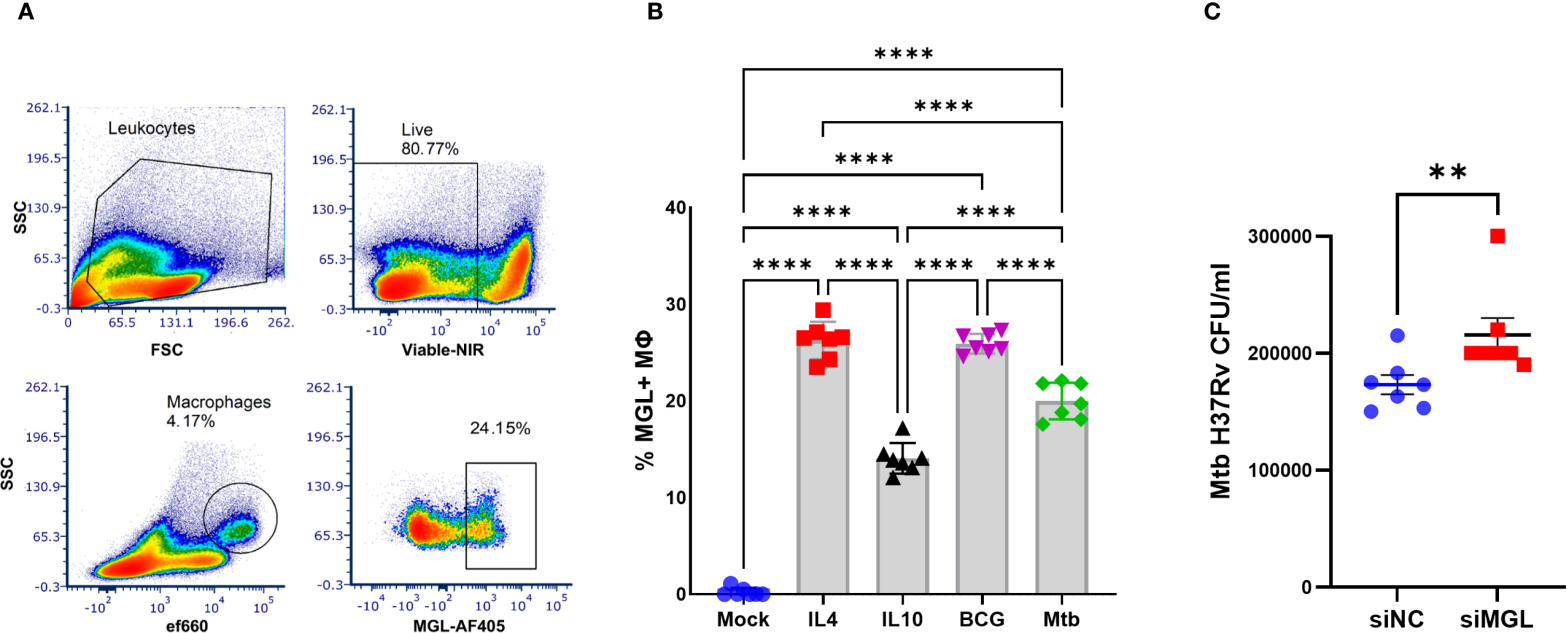
MGL expression by human MΦ is activated by mycobacteria and contributes to antibacterial function. PBMC from healthy donors were incubated *in vitro* with M2-polarizing cytokines IL-4 (100 ng/ml), IL-10 (100ng/ml) or 5 MOI of mycobacteria (BCG, Mtb) for 24 h. **(A)** MGL expression of MΦ was detected by flow cytometric analysis following selection of viable cells expressing CD11b and CD14. **(B)** Summarized results are presented as the mean ± SEM of results from 7 human donors. Statistical analysis of data with multiple groups was performed by using one-way ANOVA followed by Tukey’s test for multiple comparisons. Significant differences compared to mock treatment are indicated with ****p<0.0001. **(C)** MDM from human blood PBMC were transfected with siRNA targeting MGL (siMGL) and a control siRNA (siNC) using Lipofectamine 2000. Cells were then infected with 10 MOI of Mtb for 24 h and bacterial burden was measured in lysates following enumeration of colony forming units (CFU) after 3 weeks of growth on agar plates. Effects of RNA silencing were determined using a two tailed Student’s t-test and differences compared to mock treatment are indicated with **p<0.01.

Human PBMC from healthy blood bank donors were infected with *Mycobacterium bovis* Bacille Calmette Guerin (BCG) or Mtb and analyzed for expression of MGL by flow cytometry (**Figure 1A**). There was a significant increase in the expression of MGL on MΦ following 24 h culture with mycobacteria including both *M. bovis* BCG and Mtb. PBMC were also treated with IL-4 or IL-10 to simulate effects of M2a or M2c polarization conditions, respectively, as M2 polarized MΦ are known to induce expression of MGL (5, 20). Consistent with previous reports, both IL-10 and especially IL-4 recombinant cytokines were potent activators of MGL (**Figure 1B**) by MΦ in PBMC. Expression of MGL by human MDM was silenced using siRNA transfection (**Figure 1C**) and successful knock-down, compared to a non-targeting control, was confirmed through 96 h at both the protein and mRNA level (**Supplementary Figure S1**). Silenced cells were subsequently infected with Mtb and assessed for bacterial burden by CFU enumeration. Compared to negative controls (siNC), the cells with reduced MGL (siMGL) had a significantly higher bacterial burden (**Figure 1C**). Thus, human MGL is an important functional pathway that contributes to MΦ antibacterial activity against intracellular Mtb infection.

### MΦ MGL is activated by both M1- and M2-polarizing conditions

**Figure 2:**
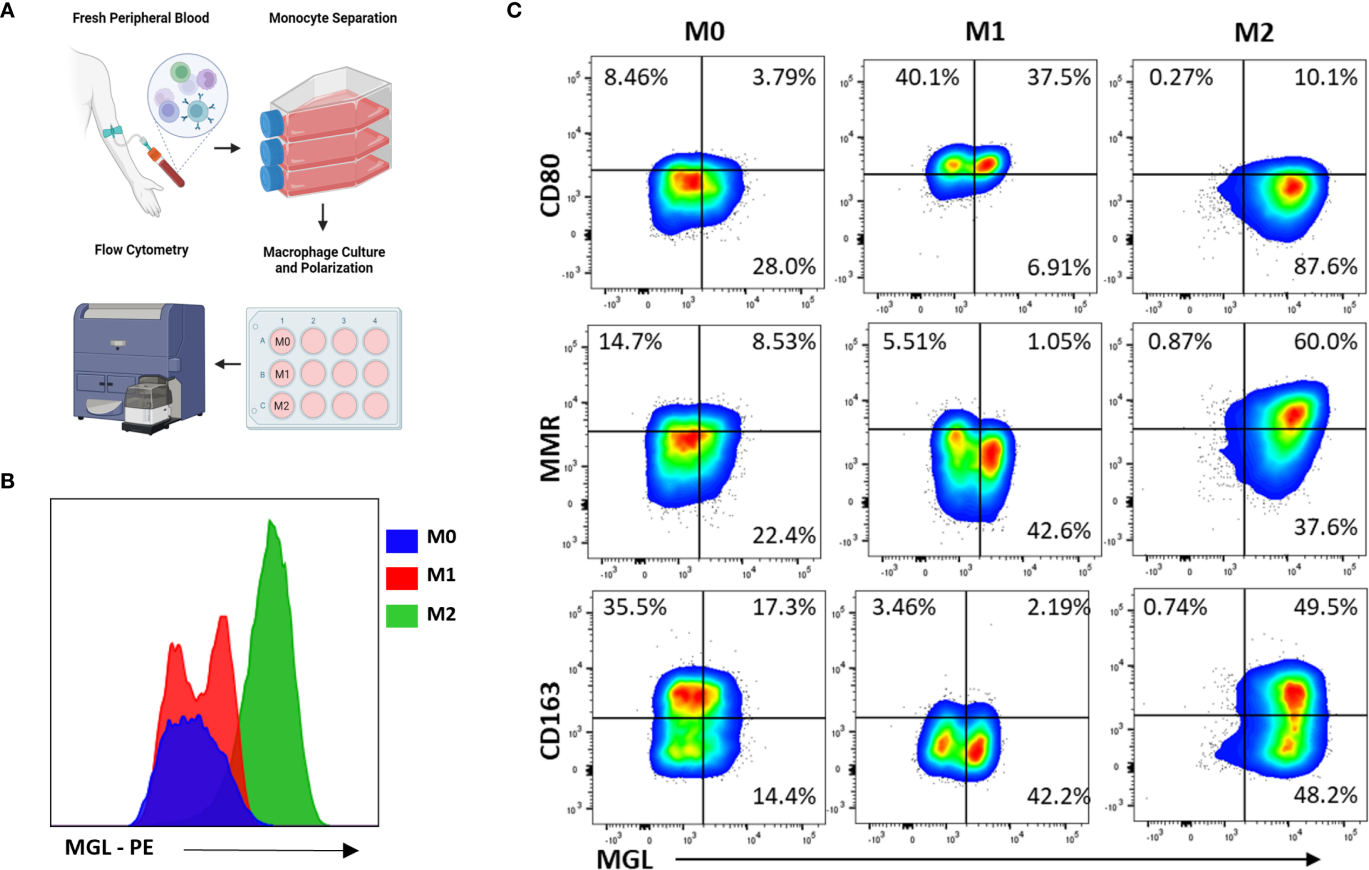
MΦ MGL is activated by both M1- and M2-polarizing conditions. **(A)** Monocytes from PBMC were separated by adherence to tissue culture treated flasks and then incubated with M-CSF for 7 days. Resulting monocyte-derived MΦ (MDM) were further incubated an additional 24 hrs in M1-polarizing conditions, IFN-γ (20 ng/mL) and LPS (100 ng/mL), or M2-polarizing conditions, IL-4 (20 ng/mL). **(B)** Cells were analyzed by flow cytometry for expression of MGL and **(C)** selected markers of polarization using untreated M0 MDM as comparison.

To demonstrate the effect of MΦ polarization on MGL expression and observe patterns of co-expression with relevant M1- and M2-asociated markers, monocytes were first separated from healthy donor PBMC and cultured in media containing M-CSF. The resulting monocyte-derived MΦ (MDM) were then treated with IFN-γ and LPS or with IL-4 to induce an M1 or an M2a phenotype, respectively, and analyzed by flow cytometry for selected polarization markers and compared to untreated M0 MDM (**Figure 2A**). As expected, MGL was abundantly expressed on M2a MΦ compared to M0 (**Figure 2B**). Interestingly, M1 MΦ also expressed MGL although at reduced levels compared to M2 MΦ (**Figure 2B**). Expression of CD80, MR, and CD163 was used to further confirm successful polarization of cells. M1 MΦ upregulated CD80 and downregulated MR and CD163 whereas M2a MΦ strongly upregulated MR but not CD80 or CD163 (**Figure 2C**). Bivariate plots comparing these markers demonstrates co-expression of MGL with MR and CD163 on M2-polarized MΦ (**Figure 2C**). In contrast, MGL co-expressed with CD80 on M1-polarized MΦ (**Figure 2C**). These results suggest that MGL co-expression with M1 and M2 markers may further define a functional phenotype or regulated state of activated MΦ.

### MGL is abundant in human TB granulomas

**Figure 3:**
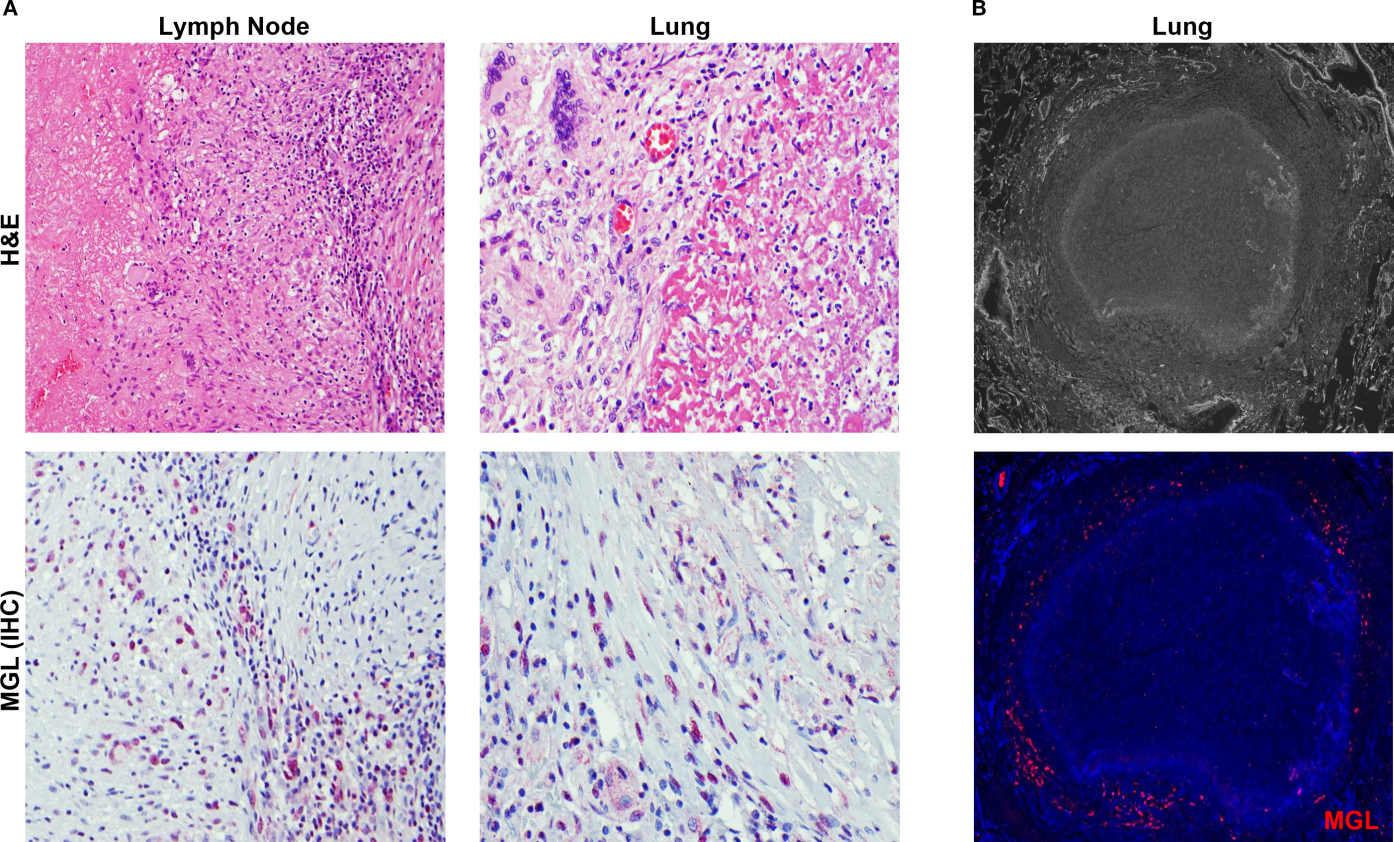
MGL is abundant in human TB granulomas. **(A)** Human tissue specimens obtained from lymph nodes at autopsy or lung biopsy were used to assess the presence of MGL+ cells in TB lesions. Tissue was analyzed following visualization of histology using an H&E stain (top panels) and immunohistochemistry (red) to detect MGL (bottom panels). **(B)** Detection of MGL in necrotic TB granuloma from autopsy specimen using epifluorescence microscopy. Positive MGL markings were detected with antibody to human MGL followed by an Alexa 555-conjugated secondary antibody (red) and DAPI to detect MGL and cellular nuclei, respectively. Sections were visualized by fluorescence microscopy at 4X (top panel) and 20X (bottom panel).

The granuloma is a pathological hallmark of TB that represents host efforts for immune containment at sites of bacterial proliferation (35). Immunohistochemical staining for MGL markings in lymph nodes (**Figure 3A**) and lungs (**Figures 3A, B**) from persons with TB revealed abundant MGL+ cells in histiocyte rich areas of inflammatory lesions. As shown in representative images (**Figures 3A, B**), cells with MGL+ markings are present within and outside the lymphocyte-rich areas in the periphery of granulomatous areas of inflammation of both necrotic and non-necrotic lesions (**Figures 3A, B**). Fluorescent microscopy analysis of a mature, necrotic granuloma in human lung specimens from autopsy demonstrated that MGL is primarily localized to the periphery of these granulomas (**Figure 3B**).

### Defects in MGL in lung and blood of PWH

**Figure 4:**
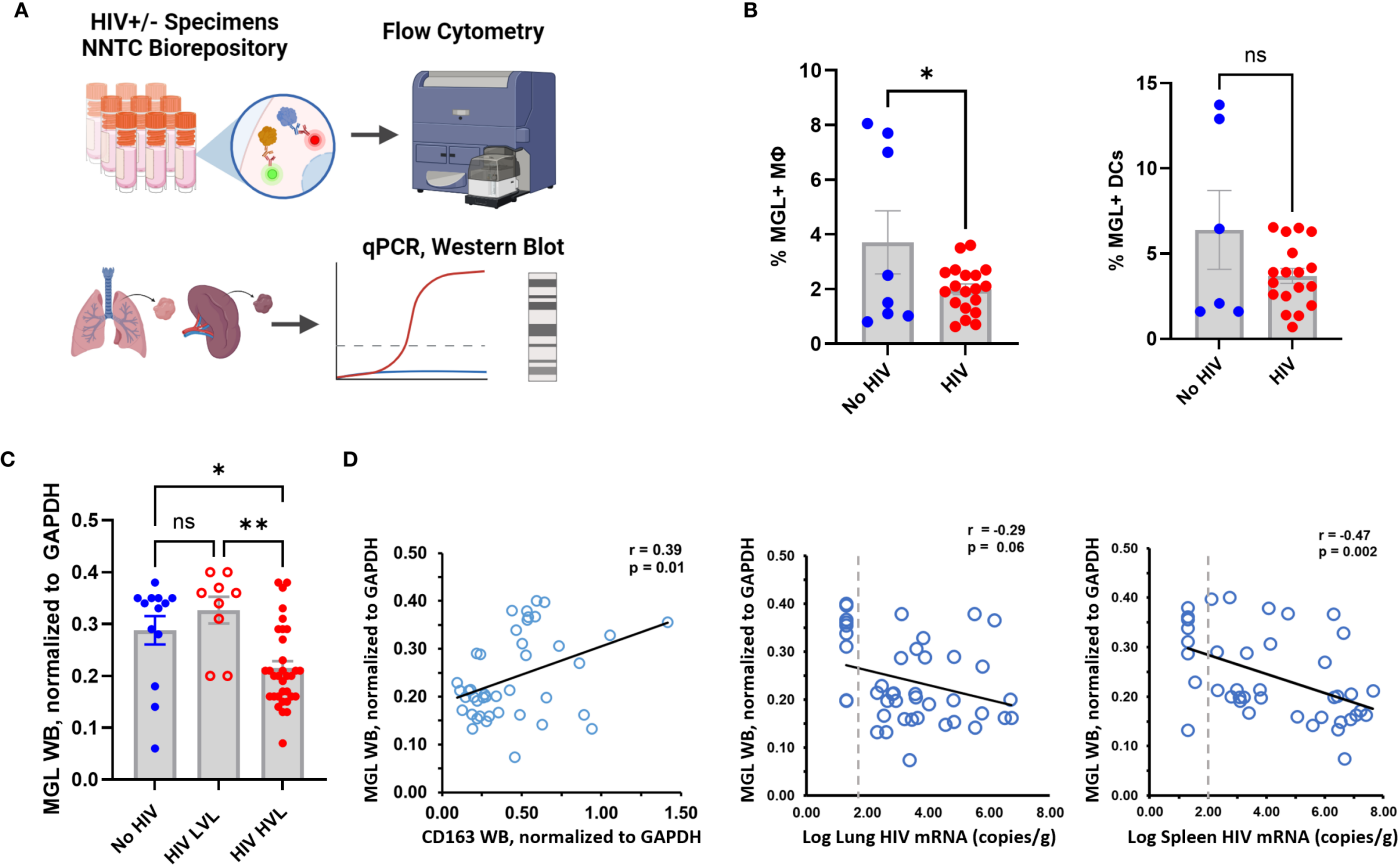
HIV status and viral load correspond with MGL defects in human lung and peripheral blood. **(A)** PBMC and tissue samples from HIV+ and HIV- donors were obtained from the NNTC biorepository and MGL expression was assessed by flow cytometry, or western blot. **(B)** Baseline expression of MGL by Mφ and DCs of human PBMC from HIV- and HIV+ donors as assessed by flow cytometry. **(C)** Western blot analysis of human autopsy tissue demonstrates reduced MGL protein in lung tissues with high viral load (HVL) of HIV and not a low viral load (LVL). **(D)** Lung MGL levels are positively correlated with lung expression of CD163, an M2 MΦ marker (left). Lung MGL levels are negatively correlated with HIV mRNA copies detected by qPCR in the lung (middle) and spleen (right). Differences among two treatments were determined using a two tailed Student’s t-test and differences compared to mock treatment are indicated with *p<0.05, **p<0.01. A Pearson’s Correlation test was used to establish relationships between viral load and MGL protein in tissue.

Immune dysregulation is observed during early phases of HIV infection. These changes include defects of the innate response that occur before severe cell-mediated immune deficiency and persist after immune restoration due to ART (3, 36–39). MΦ dysfunction due to HIV has also been reported, though the underlying mechanisms of impairment are not fully understood (40). HIV infection can modulate expression of some CLRs as a mechanism of immune evasion or viral spread including through the downregulation of MΦ MR by HIV proteins Vpr and Nef (41, 42). To determine the effect of HIV infection on MGL expression, baseline MGL levels of peripheral blood leukocytes were measured post-thaw of frozen PBMC samples from PWH (**Figure 4A**) and controls. We employed PBMC from HIV+ donors and healthy controls matched for age, gender, and race. Both MΦ and dendritic cells from PWH showed decreased MGL expression compared to HIV- donors (**Figure 4B**) although this effect varied with donor and did not reach significance in DC. We further assessed effects of HIV infection on MGL expression in the lung and determined the relationship to both lung and spleen viral load in archived human specimens. The lungs are the primary site of Mtb infection and serve as a viral reservoir for HIV even during viral suppression with ART (43, 44). The spleen is a lymphoid organ that houses a large population of latently infected CD4+ T cells during chronic HIV (45, 46). Tissue samples from decedents matched for gender, race, and age were selected from specimens in the NNTC. Selection also included non-infected control specimens as well as specimens from HIV+ decedents with and without viral suppression from ART.

Tissue viral load of ART-suppressed and non-suppressed decedents was determined in both spleen and lung using qPCR for HIV *gag.* WB of lung tissue lysates was used to determine differences in pulmonary MGL due to HIV status and relative to viral load. Compared to lung tissue from matched controls, MGL abundance was similar in HIV+ persons with low viral load (LVL) due to ART (**Figure 4C**). In lung from non-suppressed PWH that had a high viral load (HVL), MGL was decreased (**Figure 4C**). Protein levels were compared to CD163, an M2 phenotype marker, in the lungs. Consistent with reports that MGL is associated with M2 MΦ polarization, there was a moderate positive relationship (r=0.39, p=0.01) between MGL and CD163 in the lungs (**Figure 4D**). MGL protein abundance was also correlated with viral titers in the lung and spleen of the same specimen sources to determine relationships with tissue and peripheral viral burden, respectively. Results demonstrate that MGL decreased proportionally to increased viral load, as indicated by a trend toward a negative correlation (**Figure 4D**) in the lung (r= -0.29; p=0.06) and a significant negative correlation in the spleen (r= -0.47; p=0.002).

### MGL displays differential binding affinity for Mtb and HIV

**Figure 5:**
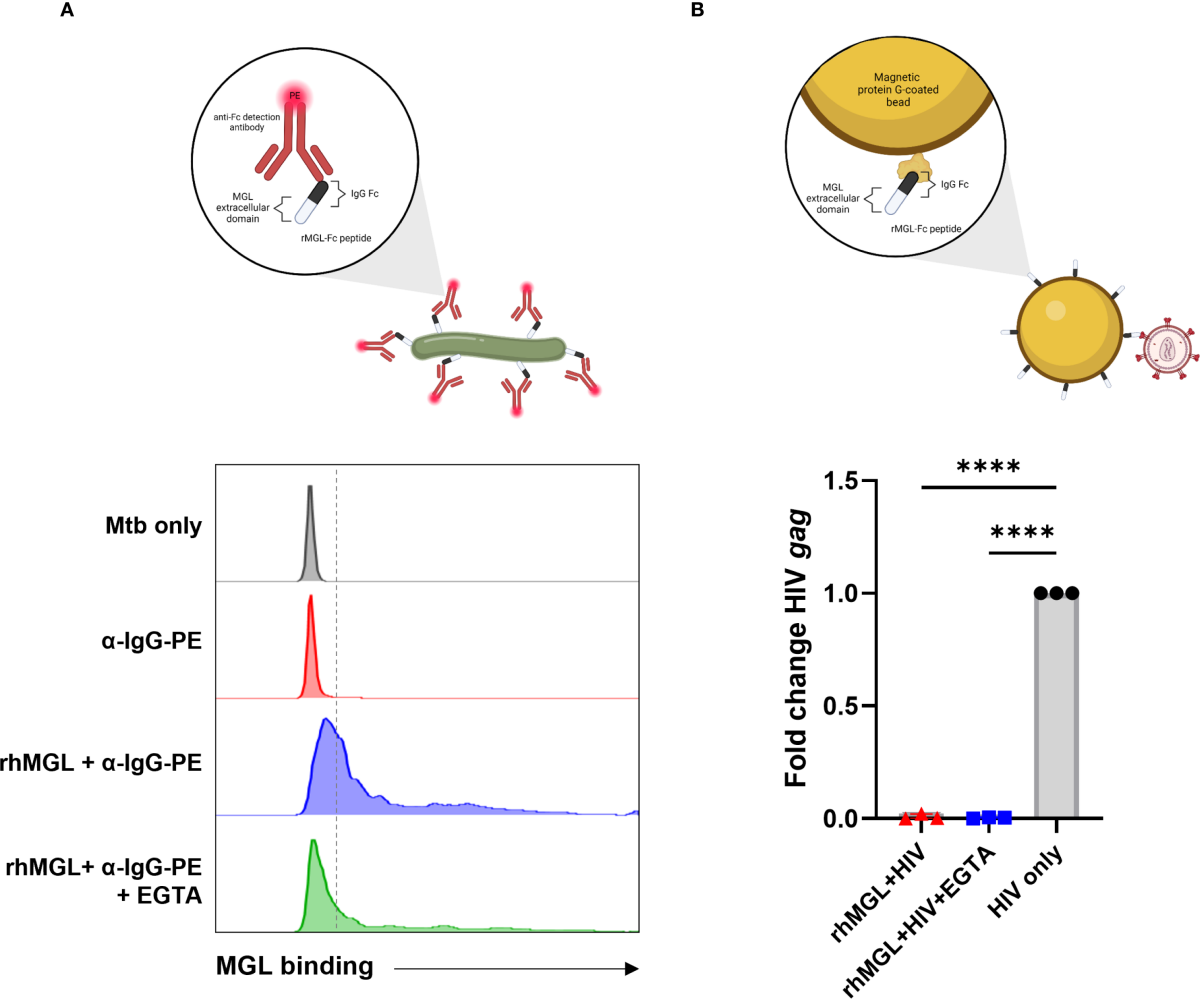
Mtb and HIV display differential binding affinity for MGL. A recombinant immunoglobulin molecule with amino acids 61–316 of human MGL expressed in the Fab region was used to determine if MGL directly binds Mtb or HIV. **(A)** Flow cytometric analysis demonstrates a strong shift in PE fluorescence upon detection of bound rMGL-Fc compared to Mtb only control or the PE-labeled detection antibody control. Inclusion of EGTA significantly reduced MGL binding to Mtb. **(B)** qPCR results from an HIV precipitation assay using rMGL-Fc immobilized to protein G Dynabeads demonstrate detection of input virus (HIV ADA, shown in black) and lack of binding to rMGL-Fc (shown in red). A negative control (EGTA) demonstrates background PCR signal upon interference with binding that is established as calcium dependent (shown in blue). Statistical analysis of data with multiple groups was performed by using one-way ANOVA followed by Tukey’s test for multiple comparisons. Significant differences among treatments are indicated with ****p<0.0001.

MGL acts as a pattern recognition receptor that recognizes carbohydrate moieties and has been shown to interact directly with some pathogens such as *Schistosoma mansoni* and *Neisseria gonorrhoeae* (47). The Mtb cell wall lacks described GalNac residues, but is heavily glycosylated, and has galactose-rich arabinogalactan structures which could be recognized by MGL (48, 49). To determine whether human MGL serves as a PRR for Mtb, a binding assay using rMGL-Fc was employed (**Figure 5A**, **Supplementary Figure S2**). This approach was used to determine whether Mtb binds to human MGL and determine the calcium-dependent nature of binding (**Figure 5A**). The interaction of MGL and Mtb (blue) was detected by flow cytometry where Mtb only (black) and Mtb incubated with the PE-labeled detection antibody (red) were used to control for presence of bacteria lacking bound antibody, auto-fluorescence, and non-specific binding of secondary antibody (**Figure 5A**). Flow cytometric analysis demonstrated a marked shift in PE fluorescence following incubation of Mtb bacilli with rMGL-Fc and detection with a PE labeled secondary reagent (**Figure 5A**). Binding was also carried out in the presence of EGTA to inhibit a calcium-dependent interaction between MGL and its ligand and to determine background noise (green). As shown in **Figure 5A**, inclusion of EGTA in the binding assay markedly reduces PE fluorescence indicative of MGL binding, consistent with the calcium-depending binding mechanism of CLRs (14). This data demonstrates direct recognition of Mtb cell wall moieties by MGL. The validity of this assay was further tested with *E. coli* strains which have been previously shown to interact with human MGL (50). *E. coli* F470 and F653 differ in LPS core oligosaccharide composition with the F470 (R1) strain containing terminal galactose which is a well-defined ligand for human MGL and the F653 (R3) lacking this ligand. The results demonstrate that rMGL-Fc binds to *E. coli* and significantly greater binding to *E. coli* with an R1 LPS core is observed compared to *E. coli* with an R3 LPS core (**Supplementary Figure S2**).

MGL has also been shown to bind viruses including Ebola and SARS-CoV-2 (51, 52), and the gp120 envelope protein of HIV is bound by various other CLRs such as Dectin-1 and dendritic cell immunoreceptor (DCIR) (37). To determine the potential for MGL to bind HIV virions, an immunoprecipitation assay using rMGL-Fc and virus as part of a bead-based isolation of Fc molecules and analysis of bound cargo was performed. This method was chosen over other immunoassays to allow binding to whole virus in the absence of a known ligand and to reduce background signal. Compared to the positive control (HIV input), limited amplification of HIV *gag* was observed following qPCR analysis of material recovered after immunoprecipitation of rMGL-Fc (**Figure 5B**). There was no significant difference in the amount of virus recovered from this method and the amount recovered when the calcium-dependent interaction was inhibited by addition of a chelating agent, EGTA, indicating lack of binding to HIV by MGL (**Figure 5B**).

### Myeloid cells in peripheral blood of HIV+ donors retain MGL functional response to Mtb

**Figure 6:**
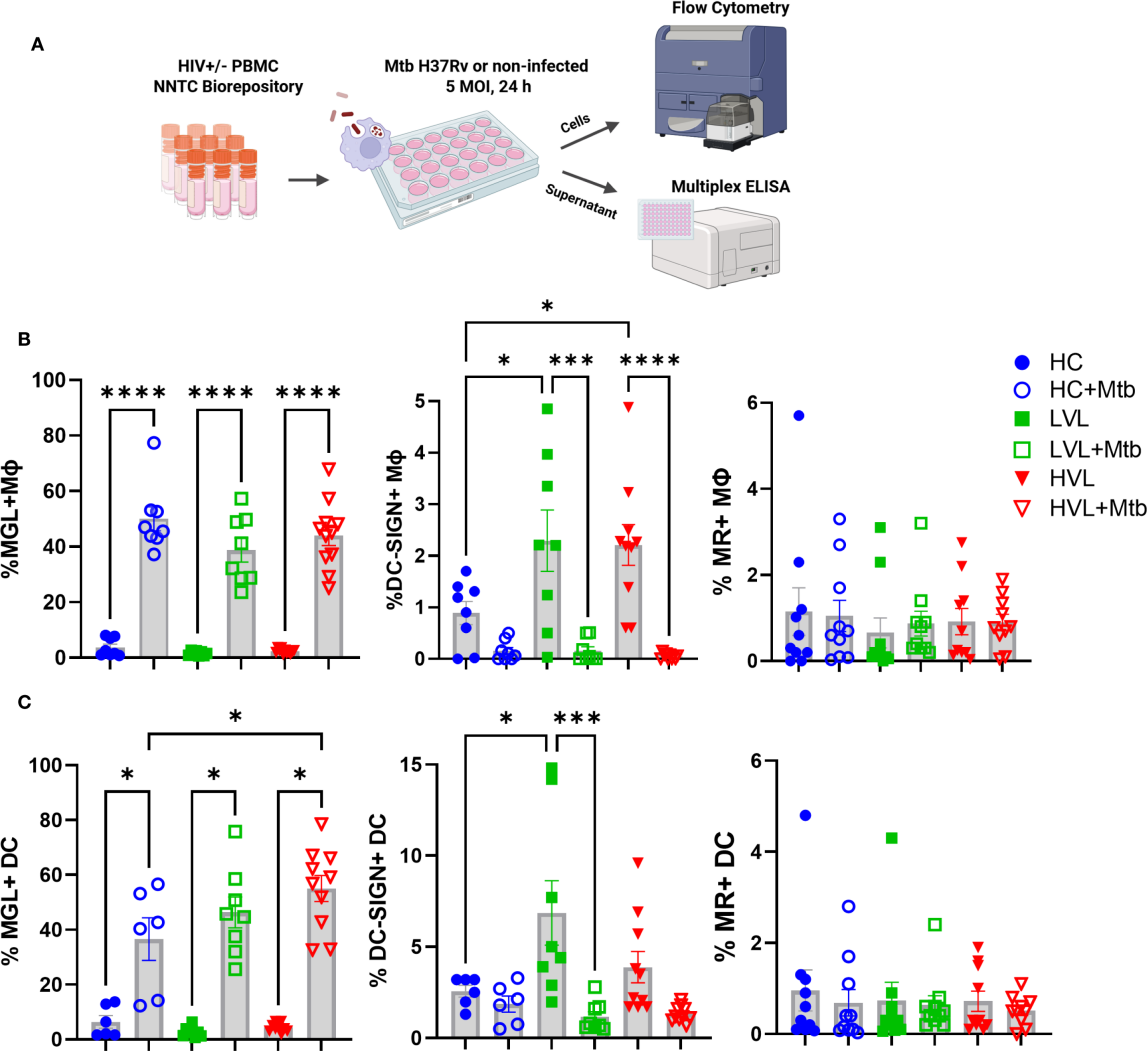
Myeloid cells in peripheral blood of HIV+ donors retain MGL functional response to Mtb. **(A)** PBMC of 19 HIV+ donors and 8 HIV- controls (HC) were exposed to Mtb H37Rv for 24 h and expression of MGL, DC-SIGN, and MR by **(B)** MΦ and **(C)** dendritic cells (DC) determined by flow cytometric analysis. HIV+ donors were further grouped based on viral load and included those with low (non-detectable) viral load (LVL, n=8) or non-suppressed donors with high viral load (HVL, n=11). Shown are summarized results of MGL, DC-SIGN, and MR, reported as percentage of live cells in the lymphocyte negative leukocyte gate. Statistical analysis of data with multiple groups was performed by using one-way ANOVA followed by Tukey’s test for multiple comparisons. Significant differences among treatments are indicated with ****p<0.0001, ***p<0.001, and *p<0.05.

To determine if PWH have a functional MGL response to Mtb, myeloid cells from archived PBMC were assessed. Thawed PBMC from HIV- controls, donors with HIV and LVL (<2 log viral copies), or HVL (>2 log viral copies) were assessed following *in vitro* culture with Mtb or mock stimuli. Following 24 h of culture, expression of MGL, DC-SIGN, and MR was measured by flow cytometry (**Figure 6A**). A marked activation of MGL in response to Mtb exposure was observed for both MΦ (**Figure 6B**) and DC (**Figure 6C**). Surprisingly, HIV status and viral load did not affect MGL expression in response to *in vitro* Mtb. In contrast, greater DC-SIGN was observed on MΦ and DC of PBMC from PWH including those with LVL and HVL (**Figures 6B, C**). Also in contrast to MGL, *in vitro* exposure to Mtb led to markedly suppressed DC-SIGN in myeloid cells of HC or PWH (**Figures 6B, C**). MR expression by myeloid cells was not affected by HIV status or *in vitro* exposure to Mtb (**Figures 6B, C**). These results demonstrate that regulation of MGL by PBMC myeloid populations differs from these well-defined CLRs with regard to HIV status and mycobacterial exposure. In view of tissue results shown in **Figure 4**, these results further suggest that HIV-mediated defects in MGL may occur through localized effects that can be overcome with appropriate activation stimuli.

### Mtb exposure differentially activates cytokines in PBMC of HIV- and HIV+ donors

**Figure 7:**
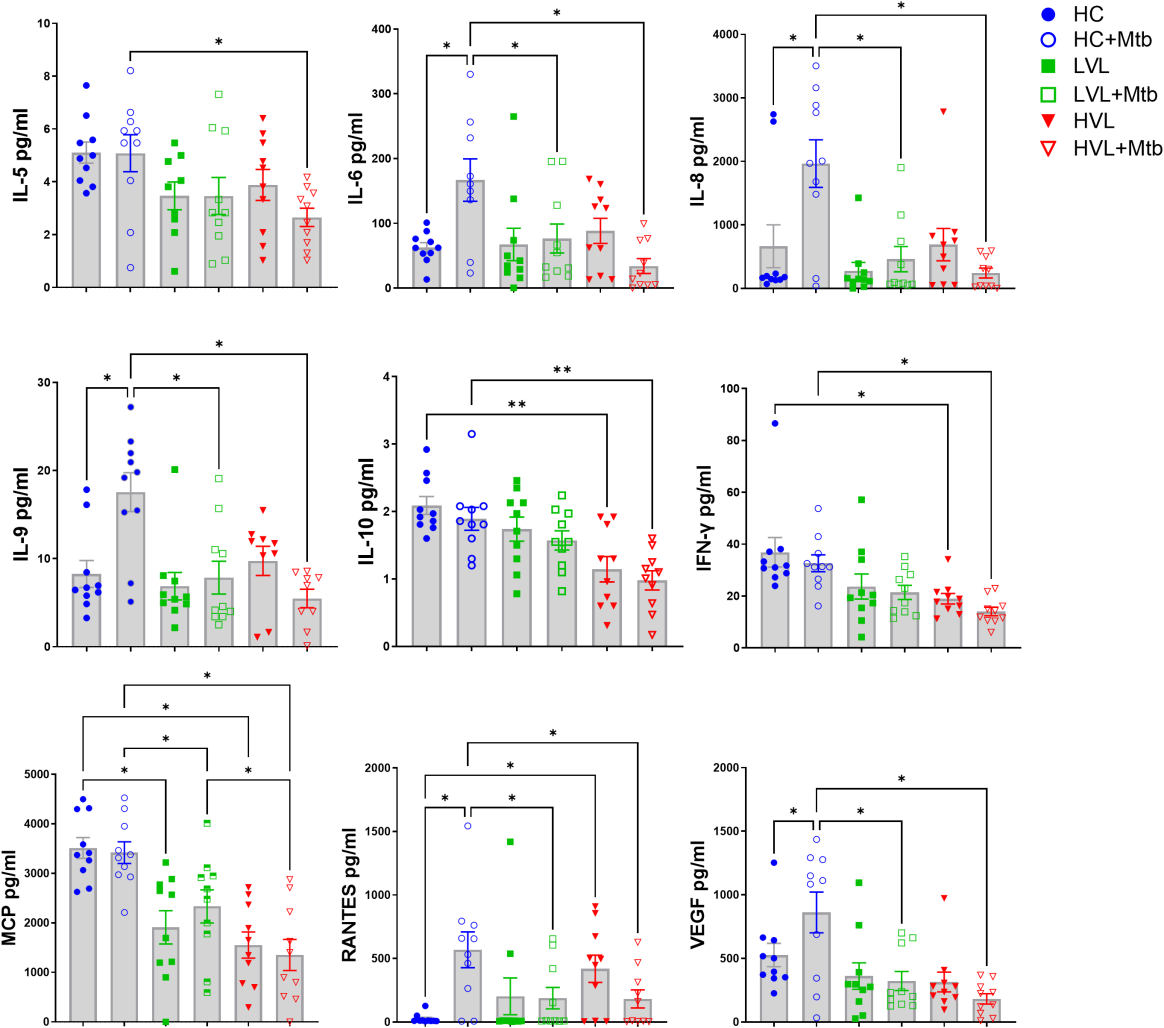
Mtb exposure differentially activates cytokines in PBMC of HIV- and HIV+ donors. PBMC of 19 HIV+ donors and 8 HIV- controls (HC) were exposed to Mtb (H37Rv) for 24 h and supernatants harvested to detect cytokine profiles (see **Figure 6A** for diagram of experimental design). Multi-plex ELISA was used to detect 27 human cytokines and chemokines in supernatants of PBMC cultured with PBS (mock) and 5 MOI of Mtb H37Rv. Cytokines which did not reach the limit of quantification were excluded from analysis. Shown are summaries of selected cytokines demonstrating differences due to HIV status and *in vitro* responsiveness to Mtb. Additional cytokines from multiplex panel are shown in **Supplementary Figure S3**. Statistical analysis of data with multiple groups was performed by using one-way ANOVA followed by the two-stage Benjamini, Krieger, and Yekutieli procedure to control false discovery rate (FDR) for multiple comparisons. Significant differences among treatments are indicated with **FDR<0.01 and *FDR<0.05.

Several cytokines (e.g., IL-4, IL-10) have been shown to activate MGL and may be a mechanism for indirect activation downstream of Mtb exposure. To determine the relationship among PBMC cytokine production in response to Mtb exposure and MGL, supernatants from the cellular experiments described in **Figure 6** were analyzed by multiplex ELISA for cytokines and chemokines commonly involved in immune responses, including during TB or HIV (**Figure 7**, **Supplementary Figure S3**).

In supernatants of PBMC from HIV- controls, Mtb exposure activated production of IL-6, IL-8, RANTES, IL-9, and VEGF. A generalized suppression of cytokine activation by Mtb due to HIV+ status was observed for these same cytokines. There was also a defect in production of IFN-γ, MCP, IL-10, and IL-5 from HIV+ PBMC compared to HIV- PBMC after exposure to Mtb, though there was not an increase in their production due to Mtb alone (**Figure 7**). The decreased MCP and IL-10 production is likely due to differences in viral load, because the cells from HVL donors produced significantly less than those from LVL donors. Interestingly, LVL PBMC did not show significant suppression of IL-5. However, there was a significant suppression in cytokine production in the Mtb*-*infected cells of HVL donors (**Figure 7**), suggesting that defects in IL-5 production may not appear until more advanced stages of HIV infection. As an important note, the PBMC donors were from a U.S. population that are not vaccinated with BCG nor have any known exposure to Mtb. These cytokine and chemokine responses thus represent innate responses to pathogen exposure and not memory recall.

There was also a significant decrease in production of IL-1ra, G-CSF, and MIP-1β and a decreasing trend in production of IL-17A, IP-10, and TNF in these cells. In PBMC from HIV- donors, Mtb exposure significantly increases production of G-CSF, IP-10, and MIP-1β (**Supplementary Figure S3**). Overall, this data suggests that HIV infection results in a suppression of many cytokines involved in immune responses to Mtb, including those known to be relevant to MGL expression.

### Subpopulation of human neutrophils express MGL that is impaired due to HIV

**Figure 8:**
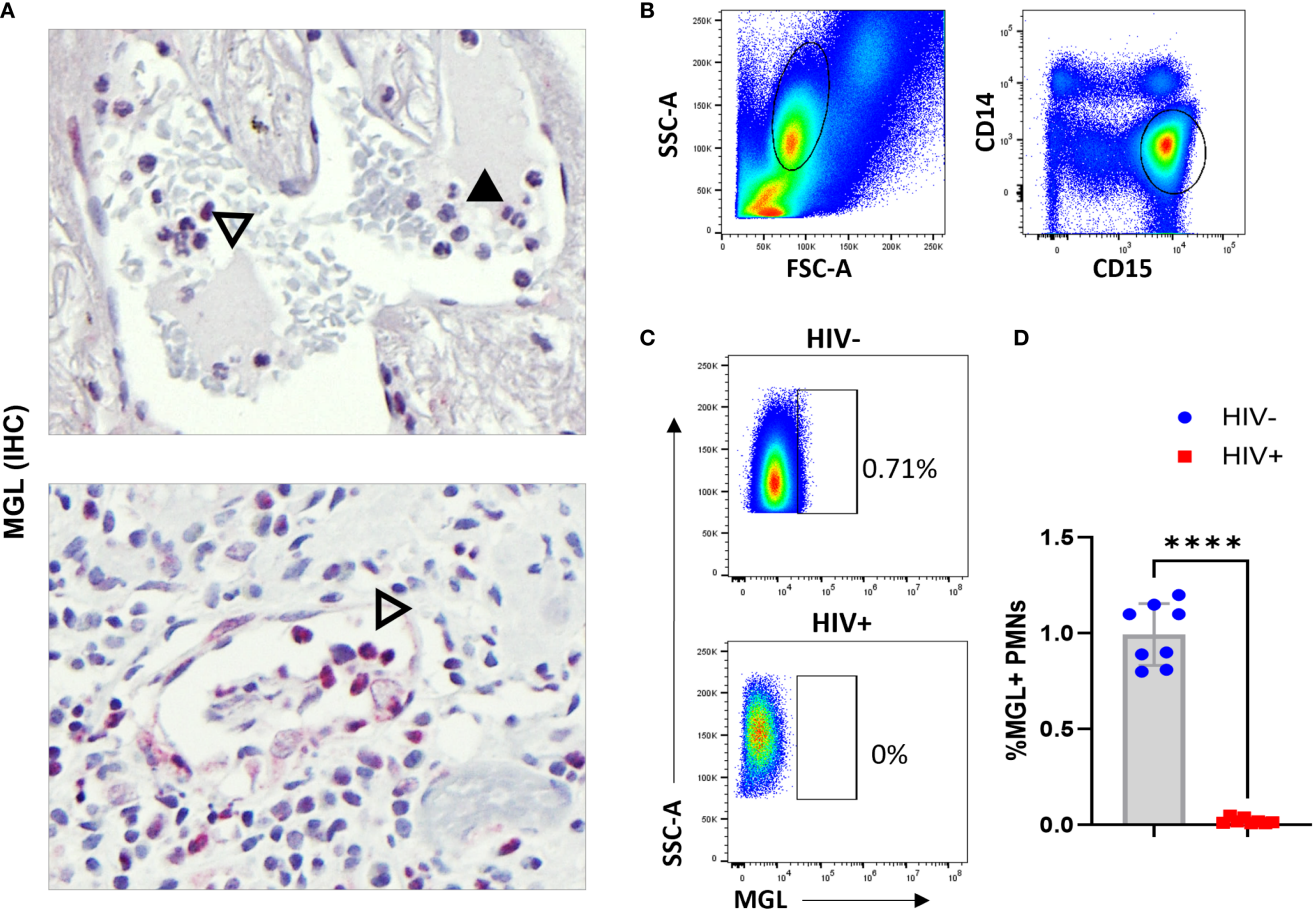
Subpopulation of human neutrophils express MGL that is impaired due to HIV. **(A)** Intravascular neutrophils in lung wedge biopsy specimen of AFB+ granuloma (top panel) and BCG bladder wall (bottom panel) containing both MGL negative (solid arrowhead) and MGL positive (open arrowhead) PMNs detected by IHC. **(B)** Human peripheral blood was isolated from HIV- and HIV+ donors and used in whole blood flow cytometric assay to detect MGL ex vivo. Shown is selection of leukocytes that excludes the lymphocyte populations and is further gated to select neutrophils based on CD14 and CD15 expression. **(C)** Detection of a MGL+ population of neutrophils in HIV- donors that is absent in blood neutrophils of HIV+ persons. **(D)** Summarized results demonstrate loss of MGL+ neutrophils in a comparison of HIV- donors (n=8) and HIV+ donors (n=8). Differences between groups were determined using a two tailed Student’s t-test and differences compared to non-infected controls is indicated with ****p<0.01.

Analysis of lung specimens for MGL expression revealed the occasional presence of cells with granulocyte morphology that were positive for MGL markings. Lung sections characterized by AFB+ granulomas contained both MGL+ and MGL- polymorphonuclear (PMN) cells. Since PMN were infrequent in the mature TB lesions in archived specimens, we explored the potential for MGL+ PMN in other relevant tissues in our clinical archive. Analysis of a bladder tissue biopsy from a person treated for bladder cancer using *M. bovis* BCG therapy revealed the presence of abundant MGL+ cells as shown near a blood vessel (**Figure 8A**). This small population of cells may be important given the role of neutrophils in the early immune response to Mtb (53). This is a potentially important observation given that it is the first report of MGL expression on a population of non-mononuclear phagocytic cells and the documented role neutrophils play in Mtb and HIV pathogenesis (54). To confirm staining specificity and determine if HIV affects expression of neutrophil MGL, whole blood (100 ul) from HIV+ and HIV- donors was used to measure MGL in neutrophils that were identified based on expression of CD14 and CD15. (**Figure 8B**). Flow cytometry measurement demonstrated that 1% of neutrophils in HIV- donors expressed MGL, a finding that represents a considerable number of cells due to the high prevalence of neutrophils in peripheral blood. When neutrophils from HIV+ donors were analyzed, however, MGL expression was markedly reduced (**Figures 8C, D**).

## Discussion

These findings identify the MGL/CLEC10A CLR as a determinant of the human PRR repertoire that orchestrates innate immunity to mycobacteria. In comparison to other CLRs that regulate immunity to mycobacteria, MGL displays unique and overlapping characteristics that are relevant to fine-tuning of the immune response. The distribution of MGL with markers that define both M1 and M2 MΦ phenotypes further supports a unique role among CLR pathways in regulating host response to infectious disease. Our observation that MGL is defective in tissues and circulating cells of PWH, but retains functional responsiveness to Mtb infection, has important implications for co-infections. Targeting MGL/CLEC10A through host directed therapies, then, may represent an avenue to boost innate immunity against mycobacterial infections while limiting inflammatory pathology.

Activation of MGL by Mtb, a pathogen associated with M1 MΦ phenotypes (55), is somewhat surprising given the association of MGL with alternatively activated (or M2) MΦ and immature DC phenotypes (34, 56, 57). We demonstrate that, similar to Mtb exposure, MGL is increased following culture with M1 polarizing stimuli (LPS+IFN-γ) although a further increase is observed in response to IL-4 (**Figure 2**). Activation of the murine homologue MGL-1 has been described following exposure to Mtb, *T. cruzi* antigens and *K. pneumoniae* (20, 28, 29). The molecular basis for activation of MGL following infections is poorly understood. We observed that primary human MΦ increase MGL in response to *in vitro* mycobacteria exposure (**Figures 1A, B**). Given the crosstalk among PRR pathways (58), it is likely that recognition of microbial ligands by one or several other PRRs promotes signaling cascades that activate the MGL promoter. Ligation of Mtb to other MΦ PRRs, including TLRs and CLRs, is described (37, 59). Alternatively, indirect activation of MGL may occur through production of cytokines that are activated by Mtb infection such as IL-4 or IL-10 (60, 61) that have been shown to activate MGL (**Figure 2B**) (34, 57).

The signaling events that results in expression of MGL on macrophages are not well characterized. Using the human monocytic cell line, U937, Katsuyama et al. demonstrated that TPA exposure induced MGL expression on macrophages in an ERK-dependent manner (62). Our observation that MGL directly binds mycobacteria bacilli (**Figure 5A**) also suggests the possibility of a positive feedback mechanism for activation of MGL gene expression. Activation of MGL in response to Mtb infection may thus be the result of direct, indirect, or combinatorial mechanisms. The molecular basis for antibacterial or immune regulatory function of MΦ downstream of MGL signaling is also poorly described. BMDM from mice lacking MGL1 display greater bacterial growth independent of changes in bacterial uptake or nitrositive stress through nitric oxide (20). Therefore, the molecular regulation of MGL and the antimicrobial pathways activated by the direct binding of mycobacteria by MGL represent important areas for further discovery relevant to development of host directed therapies.

MGL expressed by myeloid cells may also determine immune outcomes of T cells or other leukocytes. Early studies identified MGL as an immunomodulatory pathway of dendritic cells (DC) in the context of cancer (21). In line with these observations, binding of MGL to CD45 on effector T cells resulted in T cell apoptosis and decreased production of TNF and IFN-γ (24). Increased pro-inflammatory responses to pulmonary infection with Mtb or *K. pneumonia* in an MGL-1 deficient animal model further support an immune regulatory role for human MGL (28). In contrast, binding of MGL to self-antigens such as CD45RA on T regulatory cells (Treg) limited immunosuppression capabilities which restored pro-inflammatory functions of effector T cells (23). One explanation for this differential response is the demonstration that MGL activation by Tn antigens, an abnormally glycated protein overexpressed on tumor cells, promotes dendritic cell maturation and antigen presentation (22). These contradictory findings suggest a potential role of MGL in maintaining immune system homeostasis.

Our observation that MGL directly binds Mtb similar to other CLRs (e.g. DC-SIGN, MR) (63) suggests recognition of carbohydrate moieties on the mycobacterial cell wall. DC-SIGN and MR bind lipoarabinomannan and mannosylated proteins that are thought to activate innate responses by myeloid cells (64, 65). The specific GalNAc residues that define MGL binding moieties are not described for the Mtb cell wall and remain to be defined. Regardless of the ligand specificity, our findings demonstrate a functional difference between MGL and most other CLRs. Silencing of MGL limited the intracellular restriction of Mtb by human Mϕ (**Figure 1C**). These results support our previous observation that mice lacking one of two known MGL homologues displayed increased bacterial burden in the lung compared to WT littermates (20). To date, only the MΦ C-type lectin (MCL) has been described to have a similar antimycobacterial role (66) while most other CLR contribute to immune regulation or amplification of immune signaling through other PRR. Our observations that MGL+ cells localize to the outer MΦ rich zones in mature and necrotic human granulomas further suggest a role in regulation of immune responses at sites of Mtb infection including containment of Mtb proliferation (**Figures 3A, B**).

The suppressive effects of HIV on MGL expression that we describe have important implications for susceptibility to secondary infections such as Mtb in PWH. Constitutive MGL was reduced for multiple cell types in peripheral blood (**Figures 4B**, **8C, D**) as well as in lung tissue autopsy specimens of PWH (**Figures 4C, D**). In lung tissue, we identified a significant relationship between reduced MGL and increased viral burden (**Figures 4C, D**). Following *in vitro* culture, however, the functional activation of MGL by Mtb exposure in PBMC MΦ and DC was similar in PWH and age matched healthy controls (**Figures 6B, C**). Interestingly, the responsiveness of MGL to Mtb activation was independent of the innate immune suppression due to HIV that was observed in the cytokine and chemokine readouts (**Figure 7**, **Supplementary Figure S3**). These findings are consistent with the potential for local microenvironment effects due to HIV, which may include direct effects of suppressive or cytotoxic viral proteins as well as indirect effects of HIV on cellular function or survival (67, 68). In the TB granuloma, these HIV effects could be amplified due to the importance of Mϕ for Mtb containment (43) and the abundant MGL+ cells we demonstrate in the lesion zones that are associated with protection (69).

The mechanistic basis whereby HIV modulates MGL expression during acute and chronic disease or how this effect provides an advantage to the virus remains to be determined. HIV infection has negative effects on signal transduction in multiple cell types that we and others have described (12, 70, 71) that impact broad networks such as JAK/STATs (72). Another possibility is that excessive inflammation resulting from loss of MGL provides a favorable environment for HIV proliferation. HIV replication can be induced under inflammatory conditions due to the presence of binding sites for transcription factors, such as NF-κB, in the long terminal repeat (LTR) region of the viral genome (73). Alternatively, HIV targeting of MGL could serve to disable an anti-viral host defense mechanism that remains to be identified. A study utilizing a peptide mimetic of GalNAc and galactose demonstrated enhanced MΦ phagocytosis and restriction of HIV replication (74), an outcome that suggests ligation of MGL may activate anti-viral signaling. Therefore, HIV-mediated suppression of MGL could facilitate immune evasion or persistence in MΦ reservoirs. It should be noted, however, that direct interaction between the therapeutic peptide and MGL was not conclusively demonstrated as part of the previous report (74). The HIV virion is not bound by MGL (**Figure 5B**), suggesting a lack of involvement in virus uptake into mononuclear phagocytes. The effects of HIV on MGL expression that we observe indicate interference with signaling events upstream of the MGL promoter. HIV virions or viral proteins can suppress PRR and cytokine receptor signaling through multiple mechanisms (12, 75–77), offering a potential explanation for the observed defects in MGL.

Prior to our investigations, MGL was thought to be restricted to mononuclear phagocytes such as MΦ and dendritic cells (23, 47, 78). Our observation that a population of peripheral blood neutrophils express MGL (**Figure 8**) represents an important advance. Neutrophils are recruited to the lungs early after Mtb infection and have a complicated role in TB pathogenesis (79). Their phagocytic and bactericidal functions serve to restrict Mtb replication, and they produce cytokines that communicate with other cells of the immune system (80). Neutrophil activity can be a double-edged sword related to the indiscriminate effects that can damage host tissue despite antimicrobial functions (81). Although neutrophils are historically considered to be antibacterial and antifungal, it has been shown that these cells also impact outcomes of HIV infection. HIV+ individuals develop neutropenia and experience neutrophil dysfunction (54). The contributions of MGL+ neutrophils to innate immunity to bacterial pathogens and the consequences of HIV-mediated disruption warrant further investigation related to TB and other infections of PWH.

The current understanding of CLR functions during TB and HIV is informed from studies of DC-SIGN (37) and other receptors. Cumulatively, our investigations in human specimens and a murine model shows that MGL is capable of recognizing structures on the surface of Mtb (**Figure 5A**) and suppression of inflammatory responses during TB (20), similar to DC-SIGN (82). Unique among CLRs, MGL is the only receptor in the human repertoire with a preference for terminal galactose or GalNAc (83). MGL also lacks the classical ITIM or ITAM CLR signaling motifs (84), suggesting a potentially non-redundant role in immunity. HIV-induced immune defects persist despite the use of ART (85), causing individuals with controlled viral load to remain at a higher risk of developing TB (3, 85). Our findings that PBMC from donors with HIV can still upregulate MGL expression after exposure to Mtb (**Figures 6B, C**) indicate potential for this receptor to be a valuable therapeutic target in individuals with TB/HIV coinfection. This is relevant for those taking ART since HIV-induced immune defects persist despite therapy (85), and are linked with higher risk of developing TB (3, 85).

In conclusion, our results demonstrate a key role for the MGL CLR in the innate immune response by human leukocytes to Mtb infection. Our observations that HIV interferes with MGL activation in myeloid cells and neutrophils also identifies a potentially important mechanism whereby the virus could compromise antibacterial innate immunity. Understanding the role of MGL and other CLRs in immune outcomes may identify pathways that can be targeted to reduce disease progression and improve health outcomes in those with TB, HIV, or co-infections. Investigations to determine the molecular networks that regulate MGL activation in response to pathogen patterns are needed to inform our understanding of protective host immunity and development of host-directed immune modulatory therapeutics.

## Data Availability

The original contributions presented in the study are included in the article/**Supplementary Material**. Further inquiries can be directed to the corresponding authors.
