# Supplementary material for: MGL/CLEC10A is an important C-type lectin receptor activated in the innate immune response to Mycobacterium tuberculosis and is suppressed in people with HIV
Source: Front Immunol. 2025 Oct 1;16:1597281. doi: 10.3389/fimmu.2025.1597281 (PMC12522201; doi:10.3389/fimmu.2025.1597281)
Supplement: Supplementary file 1 [file DataSheet1.pdf]

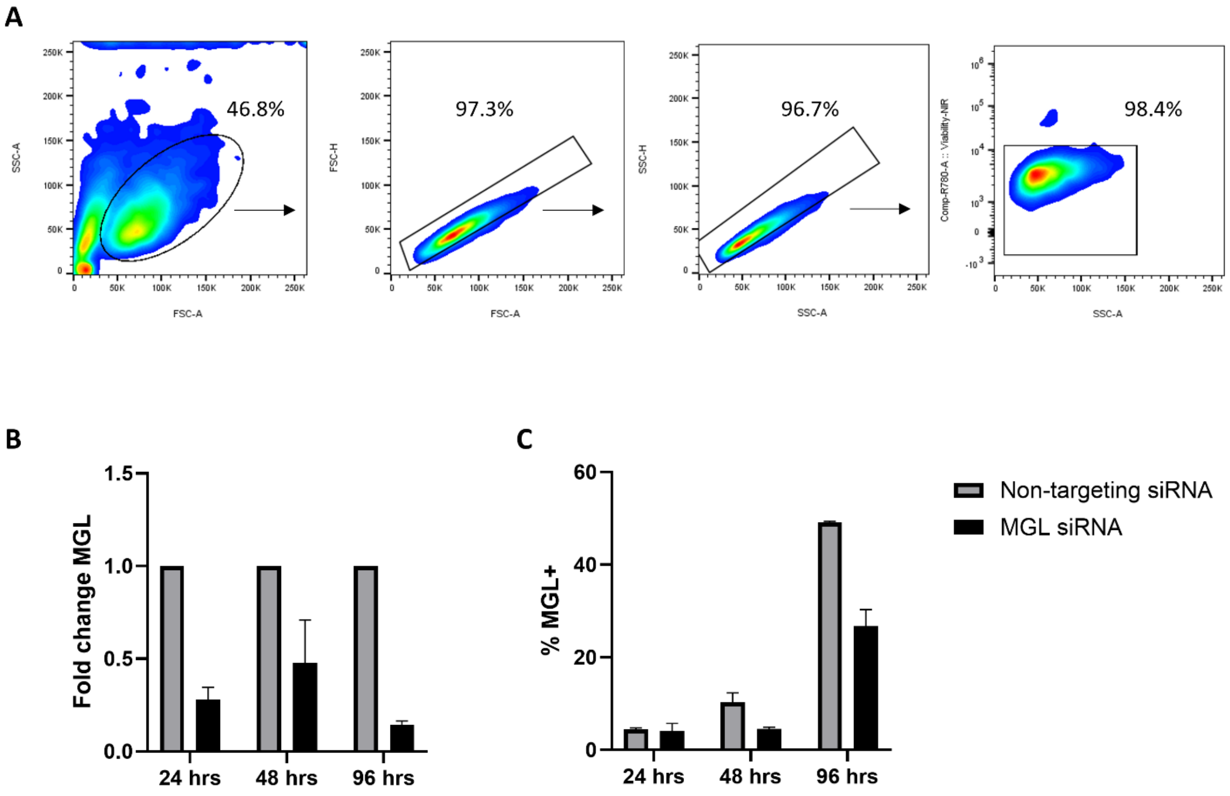

Figure S1. Surface expression of MGL decreases following siRNA mediated knockdown. (A) MDM transfected with siRNA targeting *Clec10a* were analyzed by flow cytometry to measure extracellular MGL expression. Cells were first identified based on size using FSC-A and SSC-A parameters, followed by isolation of single events in both FSC and SSC, and exclusion of non-viable cells. **(B)** Fold change of *Clec10a* mRNA was measured from samples using qPCR and calculated by $2^{-\Delta\Delta Ct}$. Expression of target gene was normalized to GAPDH and results are shown as fold change in gene expression by cells treated with anti-*Clec10a* siRNA compared to cells treated with non-targeting siRNA at each time point. **(C)** The percentage of live cells that expressed MGL was measured 24-96 hours post-transfection to confirm knockdown of MGL protein compared to cells transfected with non-specific RNA pool.

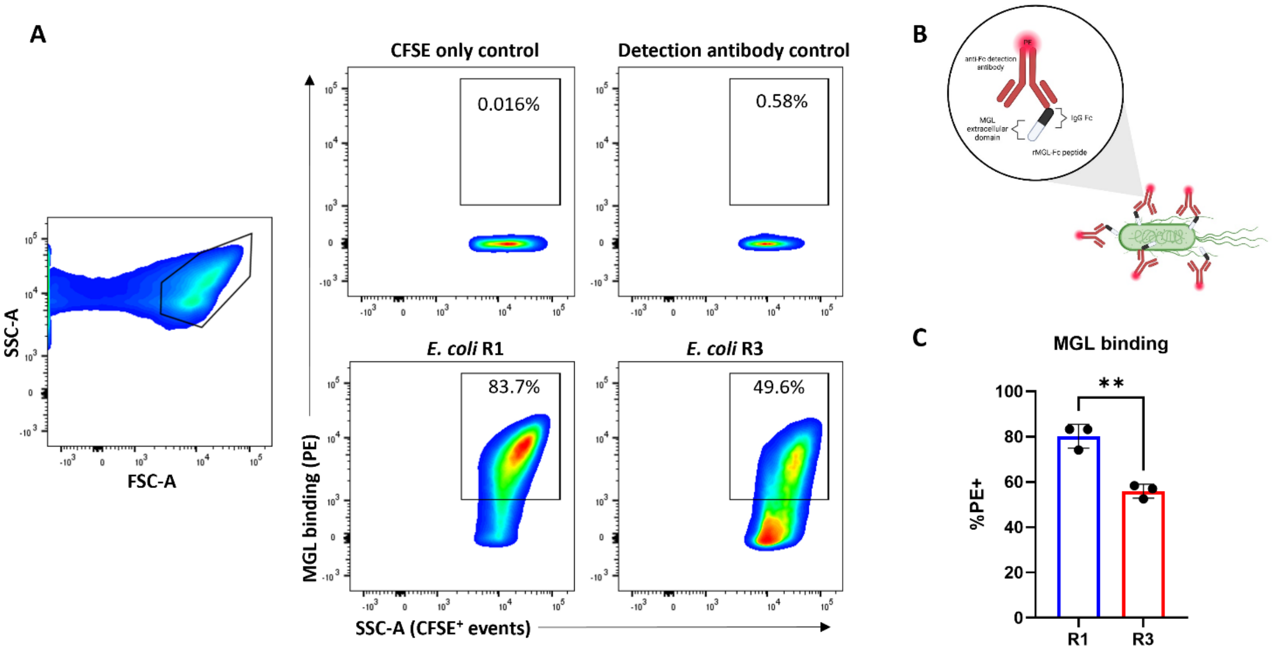

Figure S2. Differential binding of MGL to *E. coli* strains with R1 or R3 LPS polysaccharide cores. (A) Bacteria were stained with CFSE and CFSE+ bacteria analyzed by flow cytometry for MGL binding. Bacteria stained with CFSE only or with a PE-conjugated detection antibody only were also analyzed to determine spillover and non-specific binding, respectively. **(B)** Experimental design. **(C)** Summarized results of triplicate assays. The assay demonstrated greater MGL binding to *E. coli* containing an R1 LPS polysaccharide core compared to *E. coli* containing an R3 LPS polysaccharide core. Statistical significance was determined by a two-tailed unpaired t-test. ** =p < 0.01.

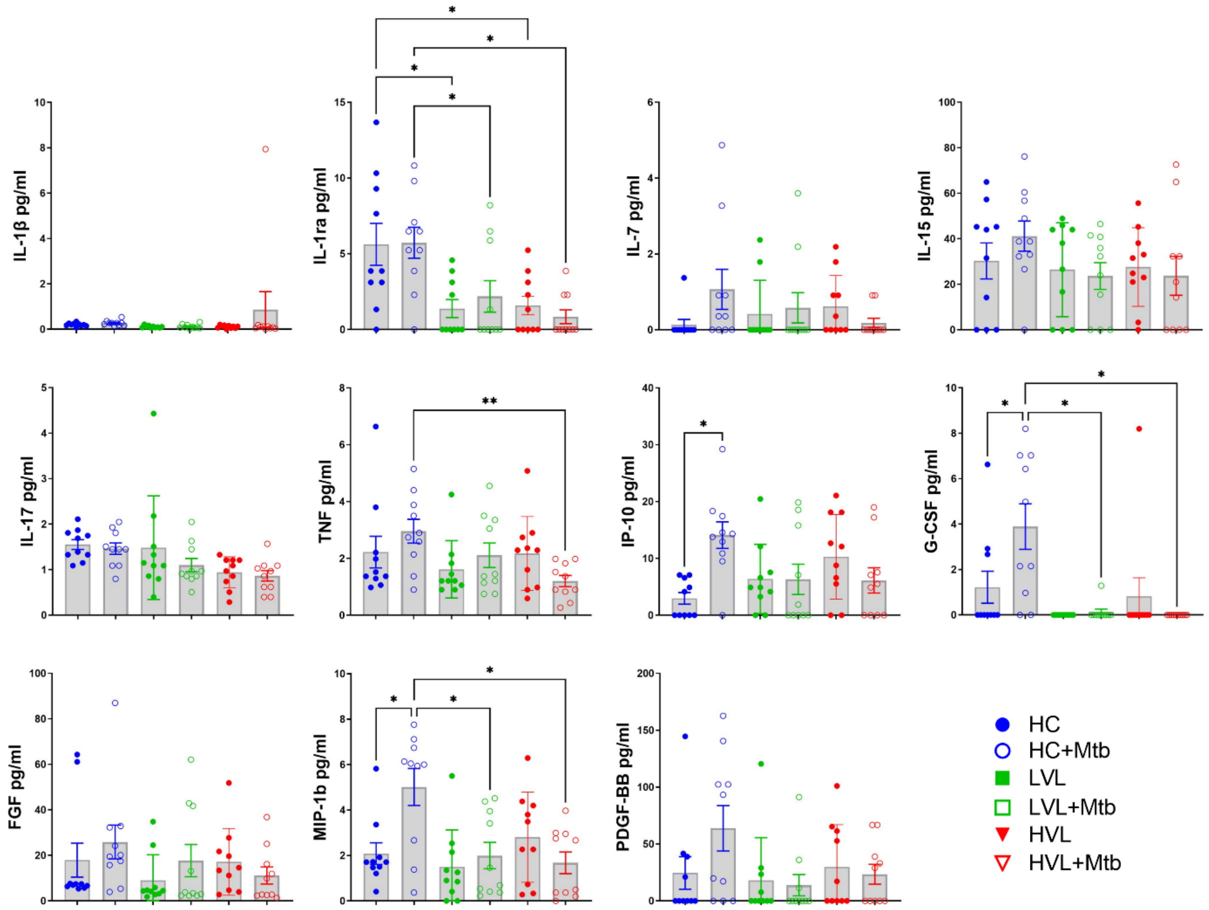

Figure S3. Mtb exposure differentially activates cytokines in PBMC of healthy and HIV+ donors (continued). PBMC of 19 HIV+ donors and 8 HIV- controls (HC) were exposed to Mtb (H37Rv) for 24 h and supernatants harvested to detect cytokine profiles (see Fig 6A for diagram of experimental design). Multi-plex ELISA was used to detect 27 human cytokines and chemokines in supernatants of PBMC cultured with PBS (mock) and 5 MOI of Mtb H37Rv. Cytokines which did not reach the limit of quantification were excluded from analysis. Shown are summaries of cytokines which were not presented in Fig 6. Statistical analysis of data with multiple groups was performed by using one-way ANOVA followed by the two-stage Benjamini, Krieger, and Yekutieli procedure to control false discovery rate (FDR) for multiple comparisons. Significance was considered with any value with an FDR < 0.05.
